# Endogenous Na^+^, K^+^-ATPase inhibitors and CSF [Na^+^] contribute to migraine formation

**DOI:** 10.1371/journal.pone.0218041

**Published:** 2019-06-07

**Authors:** Noah B. Gross, Nastaren Abad, David Lichtstein, Shiri Taron, Lorena Aparicio, Alfred N. Fonteh, Xianghong Arakaki, Robert P. Cowan, Samuel C. Grant, Michael G. Harrington

**Affiliations:** 1 Huntington Medical Research Institutes, Pasadena, California, United States of America; 2 Department of Chemical & Biomedical Engineering, FAMU-FSU College of Engineering, Florida State University, Tallahassee, Florida, United States of America; 3 Center for Interdisciplinary Magnetic Resonance, National High Magnetic Field Laboratory, Florida State University, Tallahassee, Florida, United States of America; 4 Department of Medical Neurobiology, Institute for Medical Research, Israel-Canada, The Hebrew University of Jerusalem, Jerusalem, Israel; 5 Department of Neurology, Stanford University, Palo Alto, California, United States of America; Universidade Federal do Rio de Janeiro, BRAZIL

## Abstract

There is strong evidence that neuronal hyper-excitability underlies migraine, and may or may not be preceded by cortical spreading depression. However, the mechanisms for cortical spreading depression and/or migraine are not established. Previous studies reported that cerebrospinal fluid (CSF) [Na^+^] is higher during migraine, and that higher extracellular [Na^+^] leads to hyper-excitability. We raise the hypothesis that altered choroid plexus Na^+^, K^+^-ATPase activity can cause both migraine phenomena: inhibition raises CSF [K^+^] and initiates cortical spreading depression, while activation raises CSF [Na^+^] and causes migraine. In this study, we examined levels of specific Na^+^, K^+^-ATPase inhibitors, endogenous ouabain-like compounds (EOLC), in CSF from migraineurs and controls. CSF EOLC levels were significantly lower during ictal migraine (0.4 nM +/- 0.09) than from either controls (1.8 nM +/- 0.4) or interictal migraineurs (3.1 nM +/- 1.9). Blood plasma EOLC levels were higher in migraineurs than controls, but did not differ between ictal and interictal states. In a Sprague-Dawley rat model of nitroglycerin-triggered central sensitization, we changed the concentrations of EOLC and CSF sodium, and measured aversive mechanical threshold (von Frey hairs), trigeminal nucleus caudalis activation (cFos), and CSF [Na^+^] (ultra-high field ^23^Na MRI). Animals were sensitized by three independent treatments: intraperitoneal nitroglycerin, immunodepleting EOLC from cerebral ventricles, or cerebroventricular infusion of higher CSF [Na^+^]. Conversely, nitroglycerin-triggered sensitization was prevented by either vascular or cerebroventricular delivery of the specific Na^+^, K^+^-ATPase inhibitor, ouabain. These results affirm our hypothesis that higher CSF [Na^+^] is linked to human migraine and to a rodent migraine model, and demonstrate that EOLC regulates them both. Our data suggest that altered choroid plexus Na^+^, K^+^-ATPase activity is a common source of these changes, and may be the initiating mechanism in migraine.

## Introduction

Migraine usurps the trigeminovascular pathway (meninges, trigeminal ganglion, trigeminocervical complex, thalamus, and somatosensory cortex) to cause severe headache, with widespread dysfunction extending to additional locations due to connections with the limbic system (hypothalamus, amygdala, and hippocampus) evidenced through human [1–5] and animal studies [6]. Migraine symptoms arise from altered neuronal excitability in these pathways, but the causative mechanism that triggers abnormal excitability and symptoms remains elusive [7–9]. Cortical spreading depression (CSD) [10] is strongly supported as the basis for aura in migraine [11, 12], and experimentally-triggered CSD may produce a migraine analogue [13]. The two most frequent forms of migraine are migraine with or migraine without aura. Aura may precede migraine, yet migraine-without-aura is more common [14]; twin and genetic studies suggest that migraine with aura is different from those without aura [15, 16]. Currently, CSD remains the best candidate to initiate migraine; however, what initiates CSD or other cortical triggers [17] in migraine is not understood.

Ionic disturbances are leading candidates to alter neuronal excitability in migraine [18]. Cations play important roles in regulating membrane potentials, and altered extracellular [Ca^++^], [Na^+^], and [K^+^] have been implicated in the genetic pathophysiology of several diseases, including four variants of familial hemiplegic migraine [19–22]. Polygenic gene associations in migraine are being pursued [23], however genes from these monogenic disorders do not contribute to common types of migraine [24]. Our previous studies revealed an increase in sodium concentration [Na^+^] during migraine in cerebrospinal fluid (CSF) but not in blood, whereas [K^+^], [Ca^++^], or [Mg^++^] did not fluctuate in CSF or blood [25]. Similar higher levels of CSF [Na^+^] were reported in human migraineurs compared to controls [26]. Because migraine onset is most common in early morning and late afternoon, we examined human CSF [Na^+^] over 24 h and found a 12-h rhythm that peaks at the times of most frequent migraine [27]. To study alterations of sodium homeostasis in a preclinical model, we chose the rat nitroglycerin (NTG)-triggered migraine model because NTG induces migraine in both humans [28, 29] and sensitization in rodents [30–33]. Ultra-high-field ^23^Na magnetic resonance imaging (MRI) in rats demonstrated [Na^+^] increases in the brain/CSF and eyes 20 min after intraperitoneal (i.p.) NTG administration at a [Na^+^] level sufficient to cause hyperexcitability [33]. These simulations were corroborated with studies demonstrating increased firing rates in primary cultured neurons immediately after exposure to higher extracellular [Na^+^] [34]. Together, these clinical and preclinical experiments support a role for altered CSF [Na^+^] homeostasis in migraine initiation, but evidence of direct causation is missing.

The Na^+^, K^+^-ATPase modulators, a group of steroid glycosides that include cardenolides such as ouabain and digoxin, and bufadienolides such as bufalin and marinobufagenin, have been used for centuries, and are used today to treat cardiac failure and arrhythmias in Western and Eastern medicine [35, 36]. In the last few decades, compounds similar or identical to these steroids were identified in mammalian tissues. These include ouabain [37], digoxin [38] and several bufadienolide-like compounds such as 19-norbufalin [39], 3β-hydroxy 14α 20:21-bufenolide [40], proscillaridin A [41], marinobufagenin [42] and telocinobufagin [43]. The cardiac steroid-like compounds are considered a hormone family involved in numerous physiological processes and pathological states including salt homeostasis and regulation of blood pressure, cell growth and differentiation, and behavior [44–46]. To date, the most studied cardiac steroids are the ‘endogenous ouabain-like compounds’ or EOLC. Based on immunoreactivity with anti-ouabain antibodies, these compounds have been shown to be present in mammalian brain and CSF and are considered potential neuromodulators [47, 48].

Our central hypothesis is that triggers of migraine disturb CSF [K^+^] and [Na^+^] homeostasis: inhibition of the Na^+^, K^+^-ATPase will increase CSF [K^+^] and cause CSD/aura, while its over-activation will increase CSF [Na^+^], increasing neuronal excitability, and initiate migraine [49]. Our objectives here are to search for evidence that central Na^+^, K^+^-ATPase, especially in the choroid plexus, regulates migraine, to determine whether increasing CSF [Na^+^] can cause a preclinical migraine analogue, and examine if endogenous inhibitors of the Na^+^, K^+^-ATPase are involved in CSF [Na^+^] regulation in human migraine and in an animal model of migraine.

## Materials and methods

Our study design involves both human and rodent experiments. Initial measures in human migraineurs involved testing whether endogenous Na^+^, K^+^-ATPase inhibitors are detectable in CSF, whether they change in the ictal compared to interictal states, and whether they differ from levels in CSF from controls. The animal model experiments enabled more invasive testing of CSF [Na^+^] regulation that is not feasible in humans, using nitroglycerin that can also trigger migraine in humans.

### Human study participants

The HMRI Institutional Review Board for Human Research (FWA 0000233, protocol 27197) approved the study protocol and informed consent forms. Participants were included in this study only after they gave informed and written consent. We recruited controls (N = 14) and migraineurs (N = 7, sampled twice) from 18 to 75 years of age, including both sexes, from our research clinic and from the Pasadena area through advertising, as described previously [25]. To minimize heterogeneity, migraineurs were diagnosed with migraine-without-aura, as defined by the criteria of the International Classification for Headache Disorders, 3^rd^ edition [50]. Additional inclusion criteria were: between 1 migraine per month and less than 15 headache days per month; all medications recorded for 90 d before study; no change in prophylactic medications during the 90 d; and no rescue medication taken within 48 h of sample collection. Controls were healthy individuals with no primary headache disorder, without family history of migraine in first-degree relatives, and with no prescription medication.

CSF and blood were first collected in the ictal state before rescue medication was taken. A subsequent collection was taken approximately 4 weeks later at the same time of day as the ictal state sampling when participants were in their interictal state and > 72 h from a previous migraine. The ictal or interictal state at the time of sample collection was defined as follows: 1.) The ictal state had migraine that was typical for each participant, with duration of 2 to 6 hours and severity > 5 (0 to 10 scale) at the time of CSF collection; 2.) The interictal state had no headache (0 on 0 to 10 scale) for > 72 hours. CSF was obtained by lumbar puncture using a 22-G Quincke-type needle, centrifuged (1,000g for 3 min) and supernatant stored at -80°C until thawed for EOLC assay. Venous blood was taken from the anti-cubital fossa into K_3_—EDTA Vacutainer tubes (Becton Dickinson, Franklin Lakes, NJ). Plasma after centrifugation (3,000g for 3 min) was stored at -80°C.

### EOLC determination

Extraction and determination of EOLC were performed as previously described for tissue samples [51]. Briefly, CSF or plasma samples were diluted (1:10) with methanol and centrifuged (15 min, 28,000g). The supernatant fluid was decanted and evaporated, and the dry residue was dissolved in 3 mL of PBS. The resulting solution was first separated from high molecular weight compounds, using a 3000 nominal molecular weight level membrane centrifugal filter. The lower molecular weight fraction (< 3000) containing free EOLC was diluted (1:1, vol/vol) with 0.1% trifluoroacetic acid (TFA). Each sample was loaded onto a Sep-pak C-18 column (Agilent technologies, Santa Clara, CA, USA), which was then washed with 10 mL of water containing 0.1% TFA, and the bound EOLC was eluted with 80% acetonitrile. The solvent was evaporated and the residue dissolved in PBS. EOLC levels in this solution were determined using anti-ouabain antibodies for a competitive inhibition, enzyme-linked immunosorbent assay (ELISA) as previously described [52].

### Animals

All experiments were carried out in accordance with the National Institute of Health Guide for the Care and Use of Laboratory Animals. The Institutional Animal Care and Use Committees at Huntington Medical Research Institutes (HMRI) in Pasadena, CA, and at the Florida State University in Tallahassee, FL, approved all animal procedures (protocol 28–15). A memorandum of understanding between FSU and HMRI was jointly approved. These experiments caused hyperalgesia without analgesic protection, necessary to investigate the migraine mechanism. All animals were euthanized at each experimental endpoint: immediately following behavioral testing, animals were deeply anesthetized with Somnasol (50 mg/ml solution, VetUS, Henry Schein), then transcardially perfused with PBS (0.01 M phosphate buffer, pH 7.4), followed by ice-cold 4% paraformaldehyde in PBS. After MRI procedures, animals were euthanized before waking up with CO_2_ bottled gas. One hundred and five experiment-naïve, male Sprague-Dawley rats (200–250 g) (Envigo, Indianapolis, IN) were used for all behavioral, immunohistochemical, and imaging experiments. All data were collected and analyzed by researchers without knowledge of which treatment was administered.

### Intraperitoneal procedures

Initial experiments with saline (Baxter; Passaic, NJ) gave the same behavioral and experimental responses as the NTG vehicle (buffered 30% ethanol and 30% propylene glycol) [53]; therefore, we used saline as control for NTG (American Reagent, NY). Treatments included i.p. injection of 3 mg/kg ouabain (O3125, Sigma, MO), saline (same volume), followed in 5 min by a second i.p. injection of either 10 mg/kg NTG or saline (same volume as NTG). Dosage of DigiFab (DigiFab; Savage Laboratories, NY) was tested i.p. over a wide range estimated to determine an optimal and safe level to remove nanomolar quantities of EOLC, and 0.5 mg/kg met these criteria; negative control was ovine Fab (013–0105, fab fragment; Rockland, Gilbertsville, PA).

### Intracerebroventricular (i.c.v) surgery

Rats were anesthetized with ketamine (Ketaject; Clipper Distributing Company, MO) and xylazine (Anased; Akorn, Inc., IL) by i.p. injection. Prior to surgery, the animals were given a subcutaneous injection of 0.9% saline (1 ml/kg) to prevent dehydration. A 1-mm hole was drilled through the skull (L: 1.4 mm, A: -1.0 mm) referenced to Bregma. A 24-G stainless steel guide cannula (C316GS-4/SPC; Plastics One, VA) was inserted 2.1 mm ventral to the dura mater to reach a depth of 0.6 mm dorsal to the right lateral ventricle (LV), fixed with acrylic cement, and the upper end was sealed with a dust-cap.

### Cerebroventricular infusion

Following 4–7 days from surgery, infusions were undertaken in awake rats that were held in a swathe by one investigator while a second person infused i.c.v. solutions. A 31-G internal infusion cannula (C316IS-4/SPC; Plastics One, VA) was inserted into the guide cannula to extend 1 mm into the lateral ventricle.

To test the effect of higher CSF [Na^+^], artificial cerebrospinal fluid (aCSF) solution comprised 250 mM NaCl, 2.7 mM KCl, 1.2 mM CaCl_2_, 1.0 mM MgCl_2_, 5 mM HEPES pH 7.4, 10 mM glucose, 460–480 mOsM was used. Control rats received physiological [Na^+^] in aCSF; balancing osmolarity with sucrose. aCSF osmolarity was quantified by measuring the depression of freezing point (Micro-Osmette, Precision Systems, Inc., Natick, MA).

### Behavioral testing and immunohistochemistry

Behavioral testing was performed in conscious animals in the early morning and after they were habituated to familiarity with the investigators (both males). Two tests were performed to assess behavior: aversive threshold to von Frey hairs, and relative eye closure captured by photography, as reported [33]. Immunohistochemistry of cFos was performed using published methods [33].

### MRI methodology

All scans were performed at the 21.1-T, 900-MHz vertical MRI scanner designed and constructed at the National High Magnetic Field Laboratory in Tallahassee, FL for maximal temporal resolution and spatial sensitivity [54]. The dual-tuned ^1^H/^23^Na RF probe head combines a sliding-ring birdcage coil [55] resonant at 900 MHz for the proton channel and orthogonally tuned for sodium at a resonance of 237 MHz.

Rat anesthesia was induced with 4% isoflurane in O_2_ and maintained with 3% during surgical implantation of the i.p. line, a nine-foot long section of polyethylene tubing (BPE-T10, Instech Laboratories) filled with PBS. Rats were secured in the probe vertically (head-up supine position) with monitoring of respiration and temperature. During scanning, 1.5–2.0% isoflurane was used to maintain adequate anesthetic depth, targeted to a respiratory rate between 40 and 50 breaths/min.

For simultaneous 2D ^23^Na and ^1^H multi-slice references, coronal partitions were centered at 8.5 mm anterior, 3.5 mm posterior, 6 mm posterior and 9.3 mm posterior with respect to Bregma. For anatomical localization, the placement of the MRI fields-of-view and slice packages were identified by making use of ^1^H Rapid Acquisition with Relaxation Enhancement (RARE) sequences [53]. To this end, ^23^Na CSI scans were recorded repeatedly in 9 min intervals following injection of either 10-mg/kg NTG (N = 6) or saline (N = 6) to identify alteration in total [Na^+^] relative to baseline.

CSI datasets were analyzed with an image rendering, quantification, and analysis software (AMIRA, 5.4.3, FEI Visualization Sciences Group, Boudreaux, FR). ^23^Na images were segmented via anatomical landmarks identified with aid of the scalable rat brain atlas [56]. The regions of interest included the dorsolateral 3^rd^ ventricle, cisterna magna and extracerebral sodium regions. To determine trends and significances, the 1.5-h time point ^23^Na CSI signal was converted to an absolute sodium concentration based on calibration standards and analyzed as percent change with respect to the pre-injection baseline average.

### Statistical analysis

The size of samples for both human and animal experiments were based on availability and from previous experience of the smallest number required for a reasonable enquiry of initial changes in a discovery experiment where there are no established statistics. All human and animal data was included in our analyses, and all laboratory measures were performed blinded to the treatment or ictal state to minimize bias. There was no missing CSF data, and ANOVA was used to compare CSF between ictal, interictal, and controls. Human CSF EOLC levels from migraineurs (same person in ictal or interictal state) were analyzed by a paired Wilcoxon signed rank test; migraineurs were compared to controls using Mann-Whitney. Plasma was not available from our controls; therefore we compared our EOLC plasma levels from ictal and interictal states to levels from controls in the published literature [57–62]. The mechanical aversive threshold for hindpaw withdrawal using the von Frey hair test and the trigeminal nucleus cFos expression were analyzed with a general linear model with repeated-measures analysis, using SAS v 9.2 (SAS Institute, Inc., Cary, NC). We included group (i.e. ouabain/NTG vs saline/NTG) as the between-subject factor, time as the within-subject factor, and the interaction term between time and treatment group to determine if groups differed in their withdrawal threshold. Post hoc Sidak’s multiple comparisons test was used to determine the significance of group differences at specific time-points.

## Results

### EOLC levels in CSF are low during ictal migraine

The age, sex, body mass index, and CSF total protein/cell counts for all study participants are reported in Table 1.

**Table 1:** Clinical characteristics, CSF cell counts, and total protein by diagnostic group.

	Controls	Migraineurs	P-value^1^
Number of study participants	14	7	
Age (SD) years	54 (15.7)	47 (17.9)	0.35
Women (%)	57%	71%	0.66
BMI (SD) kg/m^2^	25.2 (3.6)	23.9 (2.6)	0.58
CSF Total Protein (SD) g/L	0.349 (0.05)	0.345 (0.075)	0.9
CSF Cell Count (SD) per mL	<5 cells	<5 cells	1

^1^Fisher’s exact test for categorical variables, t-test or Wilcoxon rank sum test for continuous variables.

Controls were taking no prescription medications. All migraineurs suffered episodic migraine and were not taking prophylactic anti-migraine medication; they had not used their rescue medication within 72 hours of their interictal or ictal state assessments. Symptom ratings of migraine study participants on their two visits were distinctly different in measures of their ictal versus interictal scores, and particularly similar within each state (Table 2).

**Table 2:** Symptom rating scores of seven migraineurs in ictal and interictal state.

Self-rating scores	Ictal state mean (SD)	Interictal state mean (SD)
Pain level (0–10 scale)	8 (1)	0
HA duration (hours)	3.9 (1.3)	0
Days since last ictus	0	12.3 (8.4)
Light sensitivity (0–3 scale)	2 (0.8)	0
Sound sensitivity (0–3 scale)	1.7 (1.1)	0
Nausea (0–3 scale)	1.7 (1.1)	0

**Fig 1:**
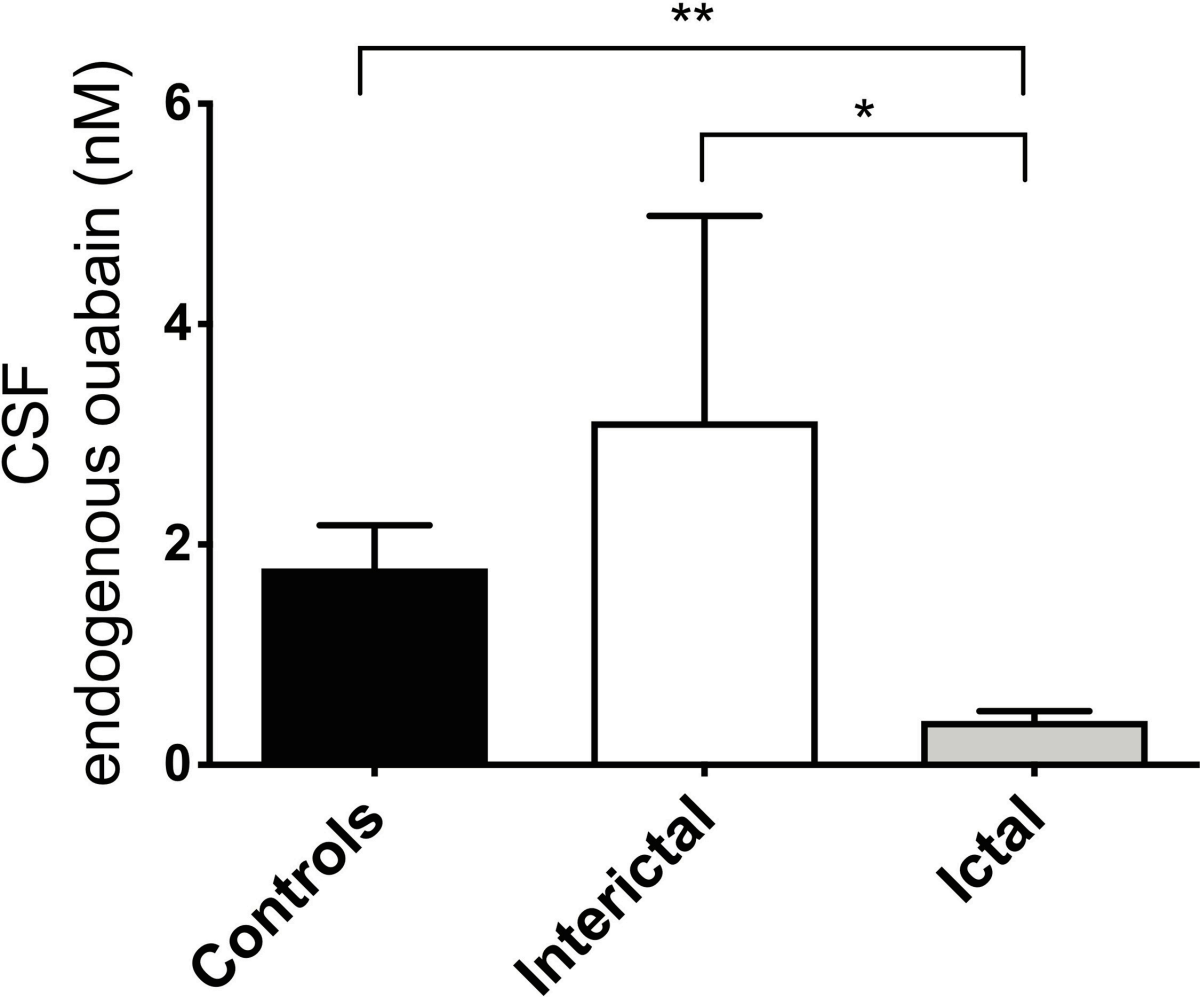
EOLC levels are lower in CSF from ictal migraineurs. EOLC levels in CSF from clinical groups were measured by ELISA using specific anti-ouabain antibodies, described in Materials and Methods. ANOVA revealed that the three groups differed significantly; ictal were lower than interictal levels (paired Wilcoxon signed rank test), and lower than controls (Mann-Whitney). * p = < 0.05; *** p = < 0.001.

CSF EOLC levels were higher in headache-free controls (1.8 nM ± 0.4) compared to ictal and interictal migraineurs, as assessed by ANOVA, p = < 0.001. EOLC levels were significantly higher in CSF during the interictal (3.1 nM ± 1.9) compared to their ictal state (0.4 nM ± 0.09), and higher in controls than in ictal migraine (Fig 1).

Plasma EOLC levels did not differ between ictal and interictal migraine states, but are higher in migraineurs in both states compared to controls [57–62] (Table 3).

**Table 3:** Plasma EOLC levels in migraineurs and controls.

Ictal migraineurs Plasma EOLC: nM (SD)	Interictal migraineurs Plasma EOLC: nM (SD)	Controls (from literature) Plasma EOLC: nM (SD)
**1.2 (1.7)**	**1.2 (1.8)**	**0.33 (0.19)**

### EOLC regulate preclinical allodynia

**Fig 2:**
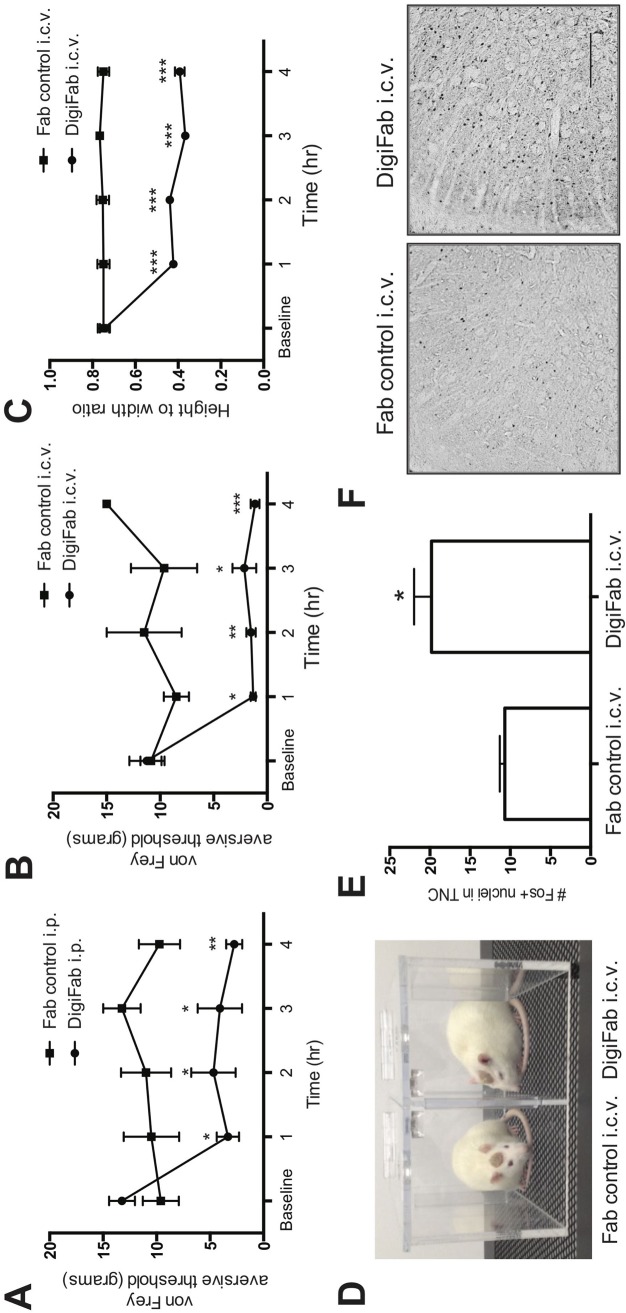
Removal of EOLC causes nociception. **(A)** Rats were treated with i.p. DigiFab or Fab control and their mechanical aversive threshold determined using the von-Frey method hourly over 4 hours. Means (SEM) displayed, n = 8–12 rats per group. **(B-F):** Rats were treated with i.c.v. DigiFab or Fab control. **(B)** The aversive threshold response to von Frey hairs is displayed as an average of both hindpaws before and for 4 hours after i.c.v. pre-treatment with Fab control or DigiFab. Means (SEM) displayed, n = 4 rats per group. **(C)** The eyelid closure response was measured before and hourly for 4 hours after pre-treatment with DigiFab or Fab control. **(D)** Representative examples of open eyes after i.c.v. Fab control (left) compared to eye squinting after i.c.v. DigiFab (right). **(E)** The number of cFos nuclei in the TNC was quantified in formaldehyde-fixed brains immediately following behavioral testing. *p < 0.05. **(F)** Representative photomicrographs of the cFos response in formaldehyde-fixed brains. Scale bar = 200 μm. * = p < 0.05; ** = p < 0.01; *** p < 0.001.

To test the hypothesis that EOLC modulates pain threshold, we treated rats with DigiFab (0.5 mg/kg, i.p.), a specific anti-digoxin antibody which sequesters EOLC [63]. Following DigiFab, the aversive pain threshold decreased across time compared to the Fab fragment control group, F (4,24) = 6.7, p = < 0.001 (Fig 2A). Post-hoc analyses ascertained that groups were not different from one another at baseline (t (24) = -1.4, p = 0.2) but differed at 1 h (t (24) = 2.8, p = < 0.01), 2hrs (t (24) = 2.5, p = < 0.05), 3 h (t (24) = 2.7, p = < 0.01) and 4 h (t (24) = 3.2, p = < 0.01) after i.p. DigiFab administration.

To localize the site of this action, rats received an i.c.v. infusion of 10 μl of DigiFab over 5 minutes, or 10 μl of negative control Fab. Our results indicate a group-by-time interaction for i.c.v. DigiFab versus Fab control treatment, F (4,22) = 6.0, p = < 0.01. Post hoc analyses revealed group differences at 1 h (t(22) = -2.52, p = < 0.05), 2 h (t(22) = -3.45, p = < 0.005), 3 h (t(22) = -2.63, p = < 0.05), and 4 h (t(22) = -4.64, p = < 0.001), but not at baseline (t(22) = 0.15, p = 0.88) (Fig 2B). In the photoallodynia measures, eye squinting in i.c.v.-infused DigiFab rats was greater than in the rats infused with the Fab control at all-time points measured, F (4,16) = 35.90, p < 0.001). Post hoc analyses disclosed group differences at 1 h (t(16) = -8.65, p < 0.001), 2 h (t(16) = -8.29, p < 0.001,), 3 h (t(16) = -10.63, p < 0.001), and 4 h (t(16) = -9.46, p < 0.001) (Fig 2C and 2D). Also similar to the effects from NTG, i.c.v. DigiFab administration causes greater TNC cFos protein expression compared to animals that received Fab fragment control infusion (F(1,6) = 5.90, p = < 0.05) (Fig 2E and 2F).

### Increasing cerebroventricular [Na^+^] recapitulates the effects of NTG sensitization

**Fig 3:**
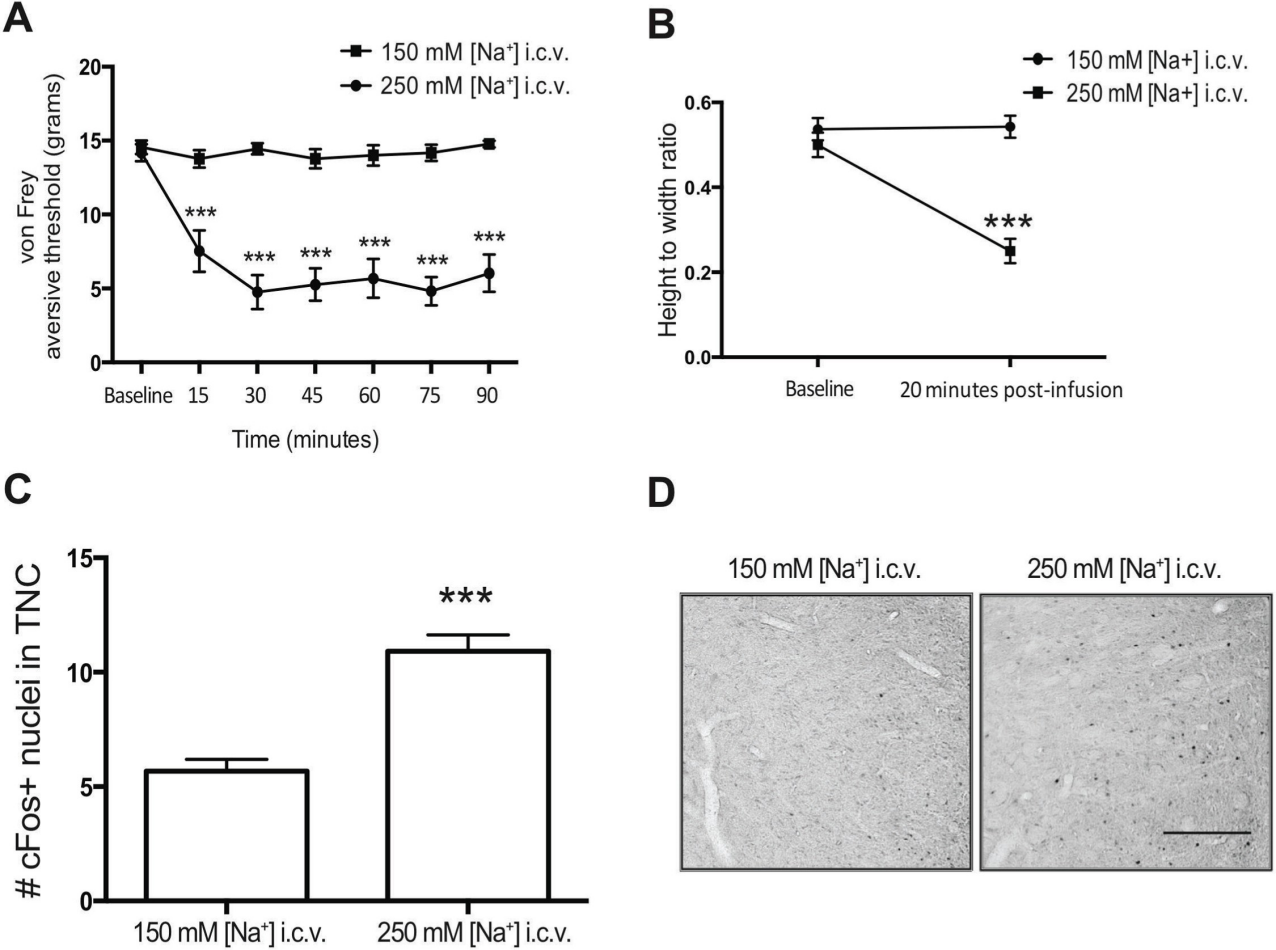
Higher i.c.v. [Na^+^] causes nociception. **(A)** The aversive threshold response to von Frey hairs was measured in both hindpaws after i.c.v. treatment with aCSF containing either 250 mM sodium (n = 14; circle) or aCSF with 150 mM [Na^+^] that was osmotically-matched to the 250 mM [Na^+^] by addition of sucrose (n = 11; square). **(B)** The eyelid closure was measured before and at 20 minutes after pre-treatment with i.c.v. 150 mM or 250 mM [Na^+^]. Means (SEM) displayed, n = 3 rats per i.c.v. treatment group. **(C)** Unilateral i.c.v. infusion of aCSF with 250 mM [Na^+^] compared to osmotically matched 150 mM [Na^+^] had increased cFos expression in the TNC. **(D)** Representative photomicrographs delineating the cFos response in the 150mM and 250 mM [Na^+^]-infused rats. Scale bar = 200 μm. (150 mM n = 10; 250 mM = 14). * = p < 0.05; ** = p < 0.01; *** = p < 0.001.

aCSF was administered to awake animals by i.c.v infusion of 2 μl/min over 5 min with either elevated [Na^+^] (250 mM; 480 mOsm) or physiological [Na^+^] (150 mM, with additional sucrose to maintain osmolarity between 470 & 480 mOsm). The withdrawal threshold with von Frey hairs indicates a group-by-time interaction for aCSF with elevated versus physiological [Na^+^] over 1.5 h, F (6,137) = 9.51, p < 0.0001 (Fig 3A). Post-hoc analyses disclosed group differences at 15 min (t (137) = -3.91, p = < 0.0001), 30 min (t (137) = -6.21, p < 0.0001), 45 min (t (137) = -5.43, p < 0.0001), 60 min (t (137) = -5.30, p < 0.0001), 75 min (t (137) = -5.99, p < 0.0001), 90 min (t (137) = -5.67, p < 0.0001), but not at baseline (t (137) = -0.27, p = 0.79). Migraine-associated photoallodynia, assessed by eye squinting (height/width ratio of eyelids) [33] was consistent with the mechanical aversion results in the same animals, with an overall group difference between elevated vs. physiological [Na^+^] assessed by one-way ANOVA, F (3,8) = 25.81, p = < 0.0005 (Fig 3B); Post hoc multiple comparisons showed increased eye squinting 20 min after infusion of the elevated vs. the physiological [Na^+^], t (8) = 7.546, p < 0.0001. These results are consistent with photoallodynia following NTG administration [64, 65].

In order to determine if increasing i.c.v. [Na^+^] produces cFos expression in the TNC similar to that observed with NTG-triggered central sensitization [31, 33], cFos was quantified in the TNC in perfused brains approximately 2 h following the [Na^+^] elevation (Fig 3C and 3D). We chose this time because 90–120 min has been reported as an early time of peak activation of TNC cFos after osmotic and chemical triggering [66, 67]. We counted more TNC cFos expression after elevated vs. physiological [Na^+^] (F (1,22) = 9.89, p = < 0.01).

### Nitroglycerin increases cerebroventricular [Na^+^] in the rat model

**Fig 4:**
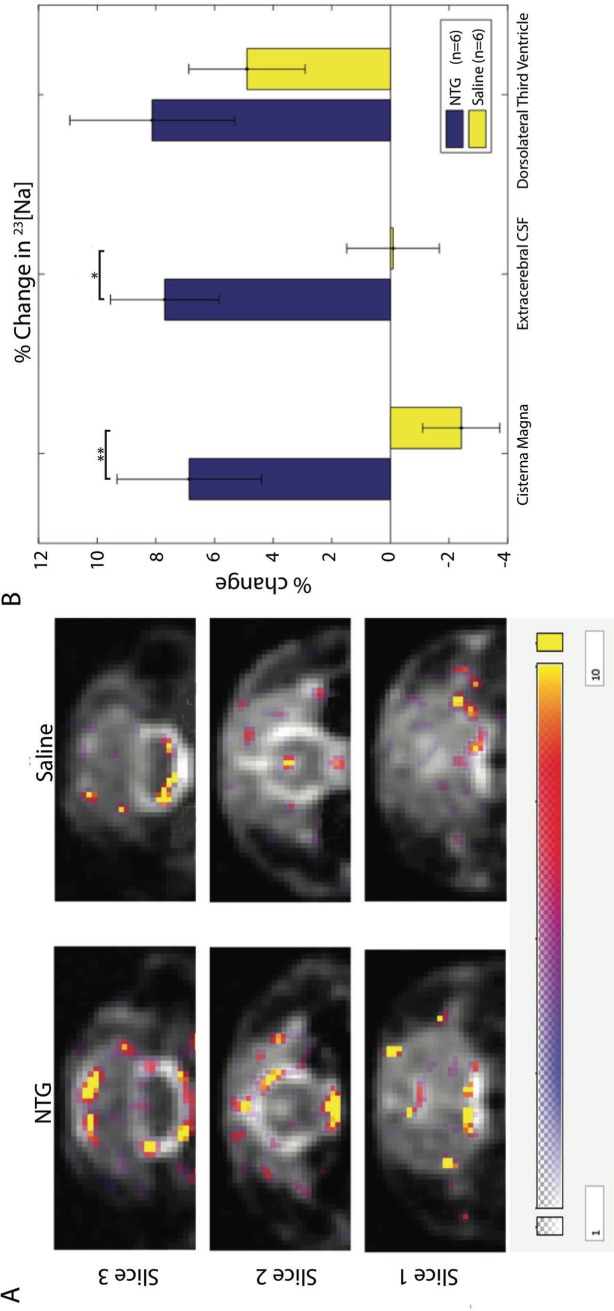
CSF [Na^+^] increases after i.p. NTG in rats. **A)** Representative anatomical coronal images for simultaneous 2D ^23^Na multi-slice CSI at 1-mm in-plane resolution after NTG (left column) and saline (right column) i.p. injections. The difference in total [Na^+^] between the pre- and post-injection (1.5-h post) time points is overlaid in color, with the color bar indicating the range of the difference in [Na^+^]. **B)** Percent changes in [Na^+^] (Mean ± SE) in three CSF regions in 12 animals. Areas of interest identified include the cisterna magna (slice 3), extracerebral CSF (slice 2), and dorsolateral third ventricle (slice 1). All regions were segmented based on a CSF concentration-based threshold. The percent change was calculated based on the calibrated 1.5-h post-injection [^23^Na] versus pre-injection baseline [^23^Na]. Significances are *p < 0.05 and **p < 0.01 (Student T-test) for comparisons between NTG and saline control.

A progressive and sustained increase of total [Na^+^] is evident in CSF compartments of anesthetized rats after i.p. injections of NTG compared to baseline: 90 min after NTG injection, [Na^+^] is distinctly increased (Fig 4), predominantly in CSF. [Na^+^] in the 3^rd^ ventricle CSF increased (not significantly), while [Na^+^] in the extracerebral and cisterna magna CSF was significantly elevated. The most pronounced change was found in the cisterna magna, where [Na^+^] increased by more than 8%, p < 0.01. The timing at 90 min after NTG of this rise in CSF [Na^+^] coincides with the earliest changes in aversive behavior, eye squinting, and cFos expression in the trigeminal nucleus caudalis (TNC) neurons from experiments with this same model [33].

### Ouabain prevents NTG-triggered central sensitization

**Fig 5:**
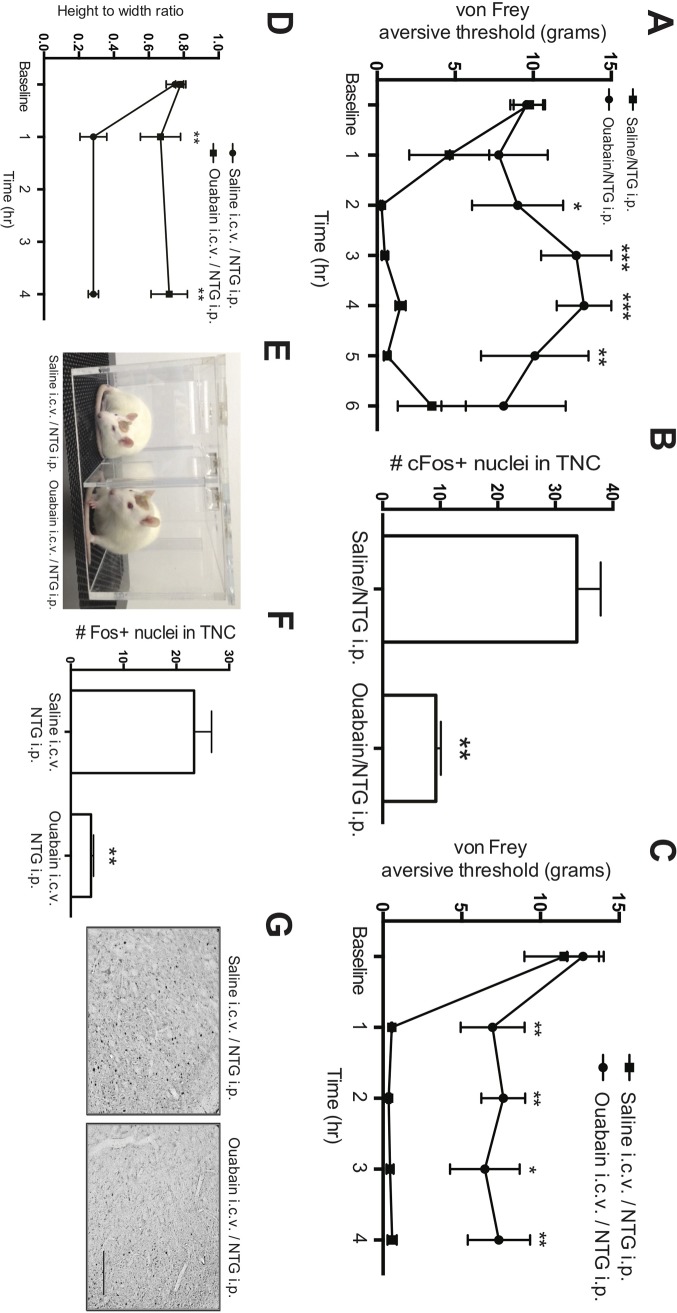
Ouabain reduces NTG-driven sensitization. Ouabain administered i.p. protects rats from i.p. NTG. The aversive threshold response to von Frey hairs was measured before and hourly for 6 hours after pre-treatment with i.p. saline (square) or ouabain (circle), followed in 5 min by i.p. NTG. Means (SEM) displayed, n = 8–12 rats per i.p. treatment group. **(B)** Saline or ouabain was given i.p. followed in 5 min by i.p NTG or saline; rats were euthanized after 4 hours, and TNC nuclei that stained for cFos were counted. **(C & D)** Pretreatment with i.c.v. ouabain administration protects against i.p. NTG treatment. **(C)** The aversive threshold response to von Frey hairs in hindpaws before and hourly for 4 hours after pre-treatment with i.c.v. ouabain or saline, followed in 10 min by i.p. NTG. **(D)** Eyelid closure at 1 and 4 hours after pre-treatment with i.c.v. saline or ouabain, followed in 10 min by i.p. NTG. Means (SEM) displayed, 3–6 per i.c.v. treatment group. **(E)** Representative examples of open eyes after i.c.v. administration of ouabain (left) compared to eye squinting after i.c.v. saline (right). **(F)** Saline or ouabain was given i.c.v. followed in 10 min by i.p. NTG administration; rats were euthanized after 4 hours, and cFos in TNC nuclei were counted. Means (SEM) displayed, n = 3–6 rats per treatment group. **(G)** Representative photomicrographs of the cFos response in formaldehyde-fixed brains. Scale bar = 200 μm. * = p < 0.05; ** = p < 0.01; *** = p < 0.005.

To test the hypothesis that specific inhibition of the Na^+^, K^+^-ATPase with ouabain protects against NTG-triggered sensitization, we pretreated rats with i.p. ouabain (3 mg/kg) or saline, 5 min before i.p. NTG (10 mg/kg), and measured the aversive threshold over 6 h. We observed that ouabain protects against the NTG-triggered reduction in aversive pain threshold, with an interaction between treatments every 30 min, F (8,48) = 3.0, p = < 0.01 (Fig 5A). Post-hoc analyses revealed group differences at 1.5 h (t (48) = 2.3, p = < 0.05), 2 h (t (48) = 2.6, p = < 0.01), 3 h (t (48) = 3.7, p = < 0.001), 4 h (t (48) = 3.6, p = < 0.001), and 5 h (t (48) = 2.8, p = < 0.01) but not at baseline (t (48) = 0.1, p = 0.9). This effect was lost at 6-h post-ouabain/NTG (t (48) = 1.2, p = 0.2). To determine if i.p. ouabain also protects against NTG-triggered neuronal expression of cFos in the rat TNC, we examined perfused brains following 4 h of behavioral assessments. Rats that received i.p. ouabain followed by NTG had less cFos expression, Kruskal Wallis, H = 9.99, p < 0.01 (Fig 5B).

To localize the site of ouabain protection in awake animals, we selected a concentration that would be below the amount required in CSF for low affinity inhibition of the Na^+^, K^+^-ATPase; we infused 10 μl of 10^−5^ M ouabain in saline vs. 10 μl of saline at 2 μl/min for 5 min, followed 10 min later by i.p. 10 mg/kg NTG administration. Ouabain administration i.c.v. protected against the aversive threshold response from NTG (Fig 5C), F (4,36) = 3.39, p = < 0.05; post-hoc analyses revealed that i.c.v. ouabain administration prevented the NTG-triggered decrease in mechanical aversive threshold at 1 h (t (36) = 2.75, p = < 0.01), 2 h (t (36) = 3.21, p = < 0.005), 3 h (t (36) = 2.55, p = < 0.05), and 4 h (t (36) = 2.94, p = < 0.01), but not at baseline (t (36) = 0.52, p = 0.60). Similar to these results, we found that the photoallodynia correlate of eye squinting was reduced in the ouabain/NTG-treated animals at all time points measured (1 and 4 h post NTG; F (2,8) = 8.92, p = < 0.01) (Fig 5D and 5E). Post hoc analyses revealed group differences at 1 h (t (8) = 3.39, p = < 0.01), and 4 h (t (8) = 3.88, p = < 0.005. Also consistent with the effects of i.p. administration, i.c.v. infusion of ouabain protected against the subsequent NTG-triggered cFos activation in the rat TNC (F (1,7) = 18.63, p = < 0.005) (Fig 5F and 5G).

## Discussion

We demonstrate, for the first time, that EOLC is protective against migraine, as evidenced by increased CSF EOLC levels during the interictal state, and decreased levels during the ictal state. This change in EOLC levels between the ictal and interictal states is independent of anti-migraine medication since their prescription usage did not change between the two sampling occasions, and they had not taken “rescue” medication before sampling. In addition, our rodent data supports cerebroventricular EOLC as a major regulator of this Na^+^, K^+^-ATPase. We also found that increasing cerebroventricular [Na^+^] sensitizes the preclinical rodent model, either by NTG injection or by directly infusing higher CSF [Na^+^]. Preventing NTG-triggered sensitization by specific inhibition of the Na^+^, K^+^-ATPase from the cerebroventricular or vascular delivery route, suggests that the choroid plexus Na^+^, K^+^-ATPase, known to be the principal regulator of CSF [Na^+^], is the most likely locus of the CSF [Na^+^] change. We report higher levels of EOLC in plasma in migraineurs compared to controls, which further support a role for altered Na^+^, K^+^-ATPase inhibitors in migraine. Plasma EOLC levels, however, do not differ between ictal and interictal states, suggesting their role reflects overall migraine biochemistry, but plasma EOLC do not protect from migraine as directly as do their CSF levels.

Trigeminal sensitization from elevated CSF [Na^+^] levels that circulate in the ventricular spaces is consistent with brain imaging that suggests the periventricular brainstem and/or hypothalamus locations may be drivers of migraine [7, 9, 68, 69]. Our studies focus on identifying the mechanism that activates these putative locations. Previous work established two routes for sodium exchange between plasma and CSF. CSF flow dynamic studies using the radioisotope ^22^Na demonstrated that the penetration of sodium into the CSF is primarily determined by a unidirectional flux from plasma across the epithelium of the choroid plexuses [70]. Four choroid plexus organs are located in two lateral, 3^rd^, and 4^th^ ventricles. Our data in this study demonstrate that the greatest rise of [Na^+^] is at the cisterna magna, supported by recent studies [53]; it remains to be investigated whether this increase arises from the 4^th^ ventricle, or from accumulation of [Na^+^] from all choroid plexuses, as CSF flows caudally. Individual ventricular choroid plexus loci may contribute differently to CSF dynamics: for instance, 55% of all choroid plexus surface epithelium in the dog is contained within the 4^th^ ventricle [71]; it will be important to determine the individual roles that each choroid plexus plays in regulating CSF [Na^+^]. Regardless, the tight junctions [72] in the choroid plexus epithelial cell layer maintain a gradient, with higher [Na^+^] and lower [K^+^] in CSF compared to blood [49], achieved primarily by the Na^+^, K^+^-ATPase [70]. Ventricular sodium diffuses rapidly with pulsatile flow (cardiac and respiratory), with some cilia-generated directionality [73–75], and rapidly enters the extracerebral and subdural CSF cavities. An additional route of exchange involves diffusion between CSF and the adjacent brain parenchyma (e.g., circumventricular organs, hypothalamus), enabled by the highly permeable ependymal lining of the ventricular cavity, which lacks the occlusive intercellular tight junctions that are characteristic of the choroid plexuses [76].

Our findings have significant scientific implications for migraine and broader pathophysiological roles for brain sodium homeostasis. Extracellular [K^+^] and intracellular [Na^+^] regulate neuron membrane channels, and their dysfunctional changes contribute to abnormal resting membrane potentials, irregular axonal conduction properties [77, 78] and neuronal hyper-excitability [34]. We propose that our results provide a mechanism whereby increased CSF [Na^+^], driven by the choroid plexus Na+, K+-ATPase, may cause increased neuronal excitability as it exits the ventricles at unmyelinated neurons, exemplified in migraine by the trigeminal and olfactory afferents in the subdural CSF regions. Lastly, it is noteworthy that the hypothalamus, which directly opposes the ventricular ependyma and is considered to be a locus of migraine initiation [69], has been shown to be the production site of brain EO [79–83].

Our data and methodology provide new insight to migraine and central sensitization, but our interpretations at this preliminary stage are limited; any generalizability will await larger studies, replication by other research groups, and comparing different diagnostic classes of headache disorder. We studied male and female migraineurs, but only male rats. These initial experiments were small and insufficient to examine common clinical confounders, including race, ethnicity, sex, age, or comorbid conditions, which will need to be studied in future experiments. More research is also needed to directly compare EOLC levels in plasma between migraineurs and controls. It will be important to extend these studies to more humans and the animal studies to include females. The immediate vasodilatory action of NTG on vascular smooth muscle precedes migraine and does not involve the Na^+^, K^+^-ATPase [84], and nitrovasodilators such as NTG inhibit the choroid Na^+^, K^+^-ATPase [85, 86]. Na^+^, K^+^-ATPase function and regulation is inherently complex, with structural subunit variations that differ across locations and cell types. The α_1_ and α_2_ isoforms are present in the choroid plexus epithelium [87], however the complexity of β and γ subunits of the Na^+^, K^+^-ATPase, and their roles in the complex and varied regulation of Na^+^, K^+^-ATPase activity requires extensive further studies, especially in the choroid [88]. Relevant in migraine, however, is that the many regulators of Na^+^, K^+^-ATPase ranging from local brain cations to adrenaline, serotonin, and estrogen are known to fluctuate in migraine [27, 49], and mutation in the ATP1A2 gene underlies FHM2 [19, 89, 90]. There is a paucity of research investigating EO in nociception, although it has been shown that inflammatory pain induced by injection of formalin is relieved by treatment with low dose ouabain and by presumed EO [91]. Ouabain was attributed to relieve inflammation in spinal pain models [92, 93], while the Na^+^, K^+^-ATPase is further involved in mu-opioid receptor analgesia [94, 95].

In summary, we achieve our primary objective and confirm that CSF [Na^+^] is regulated by EOLC in migraineurs and in NTG-sensitized rats, mediated from activated choroid plexus Na^+^, K^+^-ATPase in a preclinical model. This reveals a potential mechanism for the initiation of migraine, consistent with our hypothesis. The other arm of our hypothesis, increasing CSF [K^+^] and causing aura by inhibition of the choroid plexus Na^+^, K^+^-ATPase, remains to be tested. These different cation changes in CSF however, provide mechanisms for both aura and/or migraine, depending on how the choroid plexus Na^+^, K^+^-ATPase activity is altered beyond its homeostatic range. At this early stage in identifying the steps that initiate migraine, we suggest that EOLC has physiological roles in CSF sodium regulation, and that its homeostasis at the choroid plexus is disturbed in migraine and in the preclinical central sensitization model.

## Abbreviations

aCSF: artificial cerebrospinal fluid
CSD: Cortical spreading depression
CSF: cerebrospinal fluid
ELISA: enzyme-linked immunosorbent assay
EOLC: endogenous ouabain-like compounds
NTG: nitroglycerin
RARE: Rapid Acquisition with Relaxation Enhancement
TFA: trifluoroacetic acid
TNC: trigeminal nucleus caudalis
